# Association of the Neutrophil‐to‐HDL‐C Ratio With Coronary Artery Disease and Coronary Stenosis Severity in Patients Undergoing Coronary Angiography: Results From the Iranian‐CARDIO Study

**DOI:** 10.1155/cdr/3017486

**Published:** 2026-11-10

**Authors:** Hamidreza Mohammadi, Abbas Andishmand, Faezeh Badkoubeh, Amin Salehi-Abargouei, Mohammadtaghi Sarebanhassanabadi, Danial Chaleshi, Marzieh Azimizadeh, Matthew Budoff, Ladan Yaghmaei, Farzaneh-Sadat Mirjalili, Amin Entezari, Faezeh Golvardi-Yazdi, Mohammadreza Rafiei

**Affiliations:** ^1^ Yazd Cardiovascular Research Center Noncommunicable Diseases Research Institute Shahid Sadoughi University of Medical Sciences Yazd Iran ssu.ac.ir; ^2^ School of Medicine Arak University of Medical Sciences Arak Iran arakmu.ac.ir; ^3^ Research Center for Food Hygiene and Safety School of Public Health Shahid Sadoughi University of Medical Sciences Yazd Iran ssu.ac.ir; ^4^ Department of Nutrition School of Public Health Shahid Sadoughi University of Medical Sciences Yazd Iran ssu.ac.ir; ^5^ Nutrition Research Center Shiraz University of Medical Sciences Shiraz Iran sums.ac.ir; ^6^ School of Nutrition and Food Sciences Shiraz University of Medical Sciences Shiraz Iran sums.ac.ir; ^7^ Lundquist Institute Harbor-UCLA Medical Center Torrance California USA harbor-ucla.org; ^8^ Department of Nursing School of Nursing and Midwifery Shahid Sadoughi University of Medical Sciences Yazd Iran ssu.ac.ir

**Keywords:** angiography, biomarker, coronary artery disease, Gensini score, inflammation, neutrophil-to-HDL-C ratio

## Abstract

**Background:**

Coronary artery disease (CAD) is a major cause of morbidity and mortality worldwide. Inflammation plays a central role in the pathogenesis of atherosclerosis, with neutrophils and high‐density lipoprotein cholesterol (HDL‐C) being key components. The neutrophil‐to‐HDL‐C ratio (NHR) has recently been proposed as a potential biomarker of CAD. This study is aimed at evaluating the association of NHR with the presence and angiographic severity of CAD.

**Methods:**

This retrospective, cross‐sectional study was conducted on 250 patients who were referred for suspected CAD and underwent coronary angiography at Afshar Hospital, Yazd, Iran. Demographic, clinical, and laboratory data were collected, and the severity of CAD was assessed using the Gensini score. NHR was calculated as the neutrophil count (×10^3^/*μ*L) divided by HDL‐C (mmol/L). Multivariable logistic regression and receiver operating characteristic (ROC) analyses were performed; the model adjusted for age, sex, smoking, body mass index, and blood pressure (Model III) was designated as the primary model, and a fully adjusted model (Model IV) served as a sensitivity analysis.

**Results:**

The findings showed that patients with CAD had a significantly higher NHR than patients without CAD (3.9 ± 1.8 vs. 3.1 ± 1.28, *p* = 0.001). NHR increased across Gensini score tertiles (*p* = 0.04). In the primary adjusted model, each 1‐SD increase in NHR was independently associated with CAD (OR = 2.01, 95% CI 1.28–3.14; *p* = 0.002) and a high Gensini score (OR = 1.56, 95% CI 1.14–2.12; *p* = 0.005). Bootstrap resampling (1000 replicates) confirmed the stability of these estimates. These findings were consistent in the fully adjusted model (OR = 2.31 and 1.62, respectively). NHR showed only modest discriminative ability for CAD (AUC = 0.638) and a high Gensini score (AUC = 0.620).

**Conclusion:**

NHR was independently associated with the presence and severity of CAD in patients referred for coronary angiography. However, its discriminative ability was modest, and its incremental value and prognostic utility require validation in independent prospective cohorts.

## 1. Introduction

Coronary artery disease (CAD) remains the leading cause of death worldwide, accounting for roughly 9 million annual deaths and roughly one‐third of all deaths in individuals older than 35 years [1–3]. National data from Iran indicate that in 2019, there were 593,000 new cases of CAD, accounting for 10.3% of the total disability‐adjusted life years (DALYs) nationwide [4, 5].

Most cases of CAD result from atherosclerosis, a progressive inflammatory disease characterized by the accumulation of lipids and inflammatory cells in the arterial intima [6]. Various inflammatory cells, including mast cells, activated T lymphocytes, and macrophages, have been identified in atherosclerotic plaques [7]. Recent studies indicate that neutrophils also contribute to the process of atherosclerosis [8].

In addition, lipids and lipoproteins play a crucial role in atherosclerosis and the measurement of high‐density lipoprotein cholesterol (HDL‐C), fasting total cholesterol (TC), low‐density lipoprotein cholesterol (LDL‐C), and triglycerides (TG) is a traditional approach to assess CAD risk [9, 10]. HDL‐C, which carries approximately one‐third of plasma cholesterol, has multiple protective functions, including protection against endothelial dysfunction and reverse cholesterol transport (RCT) [11, 12]. Moreover, low HDL‐C levels are closely associated with atherosclerotic cardiovascular disease [13]. Thus, both neutrophils and HDL‐C are involved in atherosclerosis [14]. The monocyte‐to‐HDL ratio (MHR), the neutrophil‐to‐lymphocyte ratio (NLR), the triglyceride‐to‐HDL ratio (THR), the platelet‐to‐lymphocyte ratio (PLR) and the LDL‐to‐HDL (LDL/HDL) ratio have all been evaluated as markers of CAD [15–17]. The neutrophil‐to‐HDL‐C ratio (NHR) combines these two indicators; however, few studies have evaluated it [12, 17]. Therefore, in the present study, we evaluated the association of NHR with the presence and severity of CAD in patients undergoing coronary angiography (CA).

## 2. Methods

### 2.1. Study Population

This retrospective cross‐sectional study included 700 participants presenting with symptoms indicative of CAD, such as chest pain or tightness. The participants were enrolled in the Iranian‐CARDIO study conducted at Afshar Hospital, affiliated with Shahid Sadoughi University of Medical Sciences in Yazd, Iran, between July 2020 and November 2021. A comprehensive protocol of the Iranian‐CARDIO study has been previously published, providing detailed information [18]. All patients with history of coronary stent implantation, coronary artery bypass surgery (CABG), severe renal or liver disease, ongoing infection or systemic inflammatory conditions, heart failure, malignant tumors, hematologic disease, autoimmune disease, and patients with incomplete medical records were excluded. After exclusion of 287 patients who met the exclusion criteria and 163 patients with incomplete medical records, 250 patients were included in the present analysis (Figure 1).

**Figure 1:**
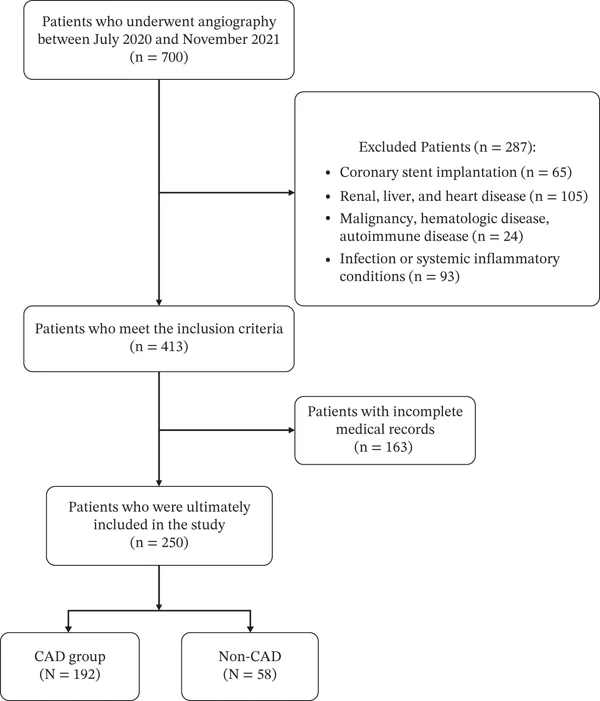
Flow chart of the study design.

All patients underwent CA through the femoral artery. The right femoral artery access was the first choice, with the left femoral artery access as an alternative. Following the procedure, patients received treatment and were subsequently hospitalized for a minimum duration of 24–48 h. Subsequently, angiographic reports of all patients were reviewed to identify cases of CAD. Patients with CAD were defined as those having ≥ 50% luminal stenosis in one or more major coronary arteries (*n* = 192), whereas patients without significant luminal stenosis formed the non‐CAD group (*n* = 58). The non‐CAD group represents symptomatic patients without significant coronary stenosis rather than a healthy control population.

### 2.2. Ethical Considerations

The study was approved by the ethics committee of Shahid Sadoughi University of Medical Sciences (IR.SSU.REC.1402.006), and the study was conducted in accordance with the Declaration of Helsinki. Informed consent was obtained from all patients prior to participation in the study.

### 2.3. Data Collection

The collected data included demographic characteristics, anthropometric measures, biochemical indices, and angiographic findings. Demographic information, including age, sex, smoking status, and history of chronic diseases such as diabetes mellitus and hypertension, was obtained using questionnaires previously used in the Iranian‐CARDIO study. Height and weight were measured using standardized procedures, and BMI was calculated as weight divided by height squared (kg/m^2^). Laboratory data were extracted retrospectively from medical records. All tests had been performed on fasting venous blood samples obtained on admission, before CA, in the central laboratory of Afshar Hospital, and included the complete blood count, FBS, lipid profile (TC, TG, LDL‐C, and HDL‐C), alanine aminotransferase (ALT), aspartate aminotransferase (AST), urea, and creatinine. The cockcroft‐gault formula was used to estimate the glomerular filtration rate (eGFR) [19]. NHR was calculated by dividing the neutrophil count (×10^3^/*μ*L) by HDL‐C (mmol/L). HDL‐C values measured in mg/dL were converted to mmol/L by multiplying by 0.02586 (equivalent to dividing by 38.67).

### 2.4. Assessment of the CAD Severity

The extent, severity, and complexity of CAD were evaluated using the Gensini scoring system. Coronary angiograms were analyzed by an experienced interventional cardiologist who was blinded to all laboratory and clinical information. Age and sex were displayed on the angiographic image header and therefore could not be masked. The Gensini score was determined by assigning severity scores to specific coronary stenoses, considering the extent of luminal narrowing and their anatomical relevance. The evaluation involved analyzing reductions in lumen diameter and the angiographic features of concentric lesions and eccentric plaques. Specific degrees of luminal narrowing (25%, 50%, 75%, 90%, 99%, and complete occlusion) were assigned scores of 1, 2, 4, 8, 16, and 32, respectively. These scores were then weighted by multiplying each lesion score by a factor corresponding to the lesion′s significance in coronary circulation. Weighting factors included 5 for the left main coronary artery, 2.5 for the proximal segments of the left anterior descending and circumflex arteries, 1.5 for the mid left anterior descending artery, 1.0 for the right coronary artery, distal left anterior descending artery, posterolateral artery, and obtuse marginal artery, and 0.5 for other segments [20, 21]. Patients with CAD (*n* = 192) were categorized into tertiles according to their Gensini scores: Tertile 1 (1–19 points, *n* = 63), Tertile 2 (20–59 points, *n* = 65), and Tertile 3 (≥ 60 points, *n* = 64). A high Gensini score was defined as the highest tertile (*n* = 64) and was compared with all other patients.

### 2.5. Statistical Analysis

The statistical analysis was conducted using IBM SPSS Version 23 (IBM Corp., Armonk, New York, United States) and Python Version 3.12 (statsmodels 0.15, SciPy 1.17, and scikit‐learn 1.8). Continuous variables were reported as the mean and standard deviation. The independent samples *t*‐test was used to assess the data between two groups, and one‐way analysis of variance (ANOVA) was conducted to find out the differences between tertiles of Gensini scores. Categorical variables are presented as frequencies and percentages and were compared using the chi‐square test. Univariable and multivariable binary logistic regression analyses were performed to examine the associations of NHR with CAD and with a high Gensini score. The findings of these analyses were reported in terms of odds ratios (ORs), along with corresponding 95% confidence intervals (95% CI). NHR was entered as a continuous variable, and the ORs are expressed per 1‐standard deviation (SD) increment (1 SD = 1.71). Four models were evaluated: Model I was unadjusted. Model II was adjusted for age and sex. Model III was adjusted for variables in Model II, along with smoking, BMI, SBP, and DBP. Finally, Model IV was adjusted for variables in Model III, in addition to FBS, TC, TG, LDL‐C, AST, ALT, eGFR, urea, DM, and HTN. Covariates were selected a priori based on clinical relevance and previous literature. Because the non‐CAD group was small (*n* = 58), Model III was considered the primary adjusted model and Model IV a sensitivity analysis. Multicollinearity was assessed using variance inflation factors (VIF), and the stability of the NHR estimates in Models III and IV was evaluated using nonparametric bootstrap resampling (1000 replicates drawn with replacement), from which percentile‐based 95% CIs were derived. Receiver operating characteristic (ROC) curves were constructed to assess the discriminative ability of NHR, and optimal cut‐off values were determined using the Youden index. A two‐sided *p*‐value of less than 0.05 was considered statistically significant.

## 3. Results

### 3.1. Baseline Patient Characteristics

The baseline characteristics of the study population are presented in Table 1. A total of 250 participants were included, comprising 192 patients with CAD and 58 patients without CAD (non‐CAD group). Patients with CAD were significantly older than those in the non‐CAD group (58.15 ± 8.99 vs. 52.65 ± 8.32 years, *p* < 0.001) and more likely to be male (*p* < 0.001). Regarding risk factors, smoking prevalence was significantly higher among CAD patients (36.5% vs. 13.8%, *p* = 0.001). However, there were no significant differences between the groups in terms of DM (*p* = 0.16) or HTN (*p* = 0.80). Laboratory tests revealed that FBS, ALT, WBC, and neutrophil counts were significantly higher in CAD patients than in the non‐CAD group (*p* < 0.05), whereas HDL‐C did not differ significantly (*p* = 0.053). Importantly, the NHR was also significantly elevated in the CAD group (3.9 ± 1.8 vs. 3.1 ± 1.28, *p* = 0.001) (Table 1).

**Table 1:** Baseline characteristics of the study population.

Variable	Non‐CAD (*n* = 58)	CAD (*n* = 192)	Total (*N* = 250)	*p*
General conditions				
Age (years)	52.65 ± 8.32	58.15 ± 8.99	56.87 ± 9.12	< 0.001
Sex, male	19 (32.8)	137 (71.4)	156 (62.4)	< 0.001
BMI (kg/m^2^)	28.01 ± 4.58	26.73 ± 4.46	27.03 ± 4.5	0.055
Risk factors				
Smoking Status	8 (13.8)	70 (36.5)	78 (31.2)	0.001
DM	14 (24.1)	65 (33.9)	79 (31.6)	0.16
HTN	29 (50)	92 (47.9)	121 (48.4)	0.8
Blood pressure (mmHg)				
SBP	125.9 ± 18.25	128.82 ± 13.75	128.14 ± 14.92	0.26
DBP	79.24 ± 10.31	79 ± 9.7	79.1 ± 9.82	0.9
Laboratory test				
FBS (mg/dL)	111.1 ± 34.4	130.6 ± 55.84	126 ± 52.25	0.002
TG (mg/dL)	149.31 ± 77.44	151.77 ± 93	151.2 ± 89.52	0.85
TC (mg/dL)	189.84 ± 78.62	201 ± 120.9	198.44 ± 112.5	0.41
LDL‐C (mg/dL)	93.72 ± 37.25	97.9 ± 42.93	96.9 ± 41.65	0.47
HDL‐C (mg/dL)	52.2 ± 14.9	48.1 ± 10.15	49 ± 11.55	0.053
ALT (U/L)	39.51 ± 36.2	63.93 ± 109.12	58.2 ± 97.67	0.008
AST (U/L)	36.76 ± 24.42	42.22 ± 32.76	40.95 ± 31	0.24
eGFR (mL/min)	86.43 ± 32.36	78.84 ± 41	80.6 ± 39.3	0.2
Urea (mg/dL)	34.5 ± 11.5	33.93 ± 9.91	34 ± 10.27	0.72
WBC (×10^3^/*μ*L)	6.86 ± 2	7.72 ± 2.05	7.51 ± 2.06	0.005
Neutrophil (×10^3^/*μ*L)	3.94 ± 1.45	4.65 ± 1.66	4.48 ± 1.64	0.004
NHR	3.1 ± 1.28	3.9 ± 1.8	3.72 ± 1.71	0.001

*Note:* Data are presented as mean ± standard deviation or number (%).

Abbreviations: ALT, alanine aminotransferase; AST, aspartate transaminase; BMI, body mass index; DBP, diastolic blood pressure; DM, diabetes mellitus; eGFR, estimated glomerular filtration rate; FBS, fasting blood sugar; HDL‐C, high‐density lipoprotein cholesterol; HTN, hypertension; LDL‐C, low‐density lipoprotein cholesterol; NHR, neutrophil‐to‐HDL‐C ratio; SBP, systolic blood pressure; TC, total cholesterol; TG, triglycerides; WBC, white blood cell.

Table 2 shows the baseline characteristics of participants stratified by tertiles of Gensini score. A higher proportion of males was observed in the higher Gensini score tertiles (low: 55.6%, moderate: 76.9%, high: 81.3%; *p* = 0.003). NHR increased progressively from the lowest to the highest tertile (3.57 ± 1.44, 3.81 ± 1.67, and 4.35 ± 2.10, respectively, *p* = 0.04). (Table 2).

**Table 2:** Baseline characteristics of the participants stratified by tertiles of Gensini score among patients with CAD.

	Gensini score			*p*
	Low (≤ 19) (*n* = 63)	Moderate (20–59) (*n* = 65)	High (≥ 60) (*n* = 64)	
General conditions				
Age (years)	57.31 ± 9.7	58 ± 8.5	59.1 ± 8.77	0.54
Sex, male	35 (55.6)	50 (76.9)	52 (81.3)	0.003
BMI (kg/m^2^)	27.05 ± 4.56	26.34 ± 4.51	26.81 ± 4.34	0.65
Risk factors				
Smoking Status	21 (33.3)	24 (36.9)	25 (39.1)	0.8
DM	15 (23.8)	27 (41.5)	23 (35.9)	0.1
HTN	29 (46)	30 (46.2)	33 (51.6)	0.77
Blood pressure (mmHg)				
SBP	128.13 ± 12.67	127.72 ± 15.45	130.63 ± 12.9	0.43
DBP	78.2 ± 8.6	79.66 ± 11	79.3 ± 9.37	0.67
Laboratory test				
FBS (mg/dL)	122.7 ± 63	132.9 ± 51.4	136 ± 52.6	0.37
TG (mg/dL)	148.52 ± 113.5	140.43 ± 71.9	166.5 ± 89.24	0.26
TC (mg/dL)	196.25 ± 101	200.62 ± 120	206.2 ± 139.9	0.9
LDL‐C (mg/dL)	92 ± 40.1	100.4 ± 43.16	101.1 ± 45.4	0.42
HDL‐C (mg/dL)	50 ± 9.7	46.7 ± 9.8	47.8 ± 10.8	0.18
ALT (U/L)	59.35 ± 98.7	74 ± 143.8	58.3 ± 73.14	0.66
AST (U/L)	37.35 ± 21.76	40.50 ± 19.35	48.76 ± 48.33	0.12
eGFR (mL/min)	79.36 ± 42	74.76 ± 20.2	82.47 ± 54.2	0.56
Urea (mg/dL)	31.74 ± 9.8	33.77 ± 11.27	36.26 ± 8	0.04
WBC (×10^3^/*μ*L)	7.57 ± 1.82	7.67 ± 2.3	7.91 ± 2	0.63
Neutrophil (×10^3^/*μ*L)	4.41 ± 1.47	4.49 ± 1.59	5.05 ± 1.86	0.06
NHR	3.57 ± 1.44	3.81 ± 1.67	4.35 ± 2.1	0.04

### 3.2. NHR Is Associated With CAD and High Gensini

In the univariable analysis, NHR was significantly associated with CAD and a high Gensini score, and these associations persisted in all adjusted models (all *p* < 0.01; Table 3). In the primary adjusted model (Model III), each 1‐SD increase in NHR was associated with higher odds of CAD (OR = 2.01, 95% CI 1.28–3.14) and a high Gensini score (OR = 1.56, 95% CI 1.14–2.12). Bootstrap resampling (1000 replicates) confirmed the stability of these estimates (percentile 95% CI 1.28–3.58 and 1.15–2.29, respectively). These findings were consistent in the fully adjusted Model IV (OR = 2.31 and 1.62; Tables S1 and S2). No relevant multicollinearity was observed (all VIF < 2).

**Table 3:** Association of NHR with CAD and high Gensini score.

Model	CAD			High Gensini		
	OR	95% CI	*p*	OR	95% CI	*p*
Model I	1.93	1.29–2.89	0.001	1.60	1.20–2.14	0.002
Model II	2.00	1.29–3.11	0.002	1.52	1.12–2.07	0.007
Model III (primary)	2.01	1.28–3.14	0.002	1.56	1.14–2.12	0.005
Model IV (sensitivity)	2.31	1.38–3.84	0.001	1.62	1.13–2.31	0.008

*Note:* The results are expressed as odds ratios (ORs) per 1‐SD increase in NHR (1 SD = 1.71) with 95% confidence intervals (CI). High Gensini: highest tertile. Model I: unadjusted. Model II: adjusted for age and sex. Model III: adjusted for variables in Model II, along with smoking, BMI, SBP, and DBP. Model IV: adjusted for variables in Model III, in addition to FBS, TC, TG, LDL‐C, AST, ALT, eGFR, urea, DM, and HTN. Estimates for all covariates in Model IV are presented in Table S1, and bootstrap results for Models III and IV are presented in Table S2.

### 3.3. Discriminative Ability of NHR for CAD and High Gensini

ROC curve analysis was performed to assess the discriminative ability of NHR for CAD and a high Gensini score. The area under the curve (AUC) for CAD was 0.638 (95% CI: 0.55–0.72), with an optimal NHR cut‐off of 2.41, yielding 83.9% sensitivity and 37.9% specificity (*p* = 0.001). For high Gensini scores, the AUC was 0.620 (95% CI: 0.54–0.70), with an optimal NHR cut‐off of 2.60, yielding 87.5% sensitivity and 30.6% specificity (*p* = 0.003). (Table 4 and Figure 2).

**Table 4:** ROC curve analysis of NHR and comparator markers for discriminating CAD and a high Gensini score.

	AUC	95% CI	Cut off	Sensitivity (%)	Specificity (%)	*p*
CAD						
NHR	0.638	0.55–0.72	2.41	83.9	38	0.001
Neutrophil count	0.626	0.54–0.70	5.47	30.7	91.4	0.002
HDL‐C	0.555	0.47–0.64	54.5	79.7	34.5	0.21
WBC count	0.624	0.54–0.70	8	46.4	77.6	0.003
High Gensini						
NHR	0.620	0.54–0.70	2.6	87.5	30.6	0.003
Neutrophil count	0.609	0.53–0.69	6.1	31.2	86.6	0.009
HDL‐C	0.537	0.45–0.62	42	32.8	76.9	0.39
WBC count	0.571	0.49–0.65	5.5	93.8	19.4	0.088

**Figure 2:**
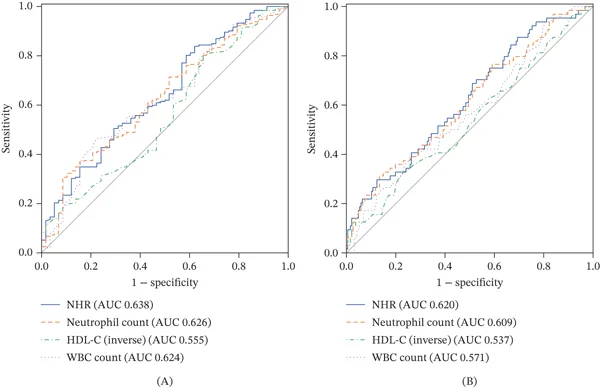
The ROC curve analysis of NHR, neutrophil count, HDL‐C, and WBC count for (A) discriminating CAD and (B) a high Gensini.

## 4. Discussion

The present study evaluated the relationship between NHR and the severity of CAD in angiography candidates. NHR was higher in patients with CAD and in those with higher Gensini scores. After adjusting for potential confounders, NHR remained independently associated with both CAD and a high Gensini score. However, NHR showed only modest discriminative ability.

CAD is a major pathology associated with all‐cause morbidity and mortality worldwide [22, 23]. Overall, early diagnosis of CAD is essential for preventing disease progression and reducing mortality rates in the coming years. In patients presenting with chest pain, evaluating the severity of coronary stenosis is essential for determining optimal treatment strategies, stratifying cardiovascular risk, and assessing the prognosis of those with CAD [24, 25]. The pathophysiology of CAD is complex, involving processes such as dysregulated lipid metabolism, inflammatory responses, oxidative damage, and other mechanisms, with inflammatory factors playing a central role [26, 27]. Plasma inflammatory biomarkers have been evaluated in patients with cardiovascular disease and found to be significant prognostic factors for both short‐term and long‐term outcomes [28, 29]. Composite ratios of hematological and lipid parameters have been proposed to provide more comprehensive information than single parameters [30–32].

Our findings revealed a significant association between the NHR and CAD, potentially attributable to the role of inflammation in the pathogenesis of atherosclerosis, a process in which circulating neutrophils actively participate [33]. Neutrophils infiltrate the vasculature in the initial stages of atherosclerosis [34]. A high neutrophil count is significantly associated with increased arterial wall stiffness and an elevated risk of ACS. Advanced atherosclerotic plaques are characterized by a heightened presence of neutrophils, with their counts showing a positive correlation to the histopathological features associated with lesions that are prone to rupture. Neutrophils drive the progression of atherosclerosis and contribute to plaque destabilization. Additionally, neutrophil aggregation and infiltration of inflammatory cells, along with platelet aggregation, lead to vascular stenosis and blockage [8]. Neutrophils play a pivotal role in acute tissue damage by secreting inflammatory mediators [35], primarily through the release of pro‐inflammatory cytokines and reactive oxygen species, which drive the inflammatory response. This process may also impair nitric oxide utilization, contributing to endothelial dysfunction and the subsequent development of atherosclerosis [36]. Furthermore, studies indicate that neutrophil count is a stronger predictor of cardiovascular events and long‐term mortality than monocyte or lymphocyte counts [15, 37]. Consistent with the studies by Chen et al. and Gao et al. [17, 38], we found that the neutrophil level in the CAD group was significantly higher than that in the non‐CAD group.

HDL‐C, as a typical lipid‐related biomarker, plays an important protective role in atherosclerosis and inflammation. HDL‐C plays a crucial role in RCT. Additionally, HDL‐C can enhance angiogenesis in endothelial cells and inhibit apoptosis in cardiomyocytes, thereby exerting cardioprotective effects [39]. Several HDL‐associated molecules, including apolipoprotein L‐1 (ApoL‐1), sphingosine‐1‐phosphate (S1P), immunoglobulins, and complement components, are directly involved in modulating the immune response [40, 41]. Moreover, HDL‐C exhibits anti‐inflammatory properties, as evidenced by its ability to reduce neutrophil activation, adhesion, and migration. This underscores the significant and complex interplay between neutrophils and HDL‐C [42]. In contrast, activated neutrophils can mediate HDL‐C oxidation and impair cholesterol efflux because of their enzymes that generate oxidants [43]. A previous study identified a reciprocal regulatory interaction between HDL‐C and neutrophils, indicating that these entities can modulate each other′s functions [28].

Several studies have reported that NHR performed better than other indicators such as NLR, PLR, MHR, THR, and LDL‐C/HDL‐C in predicting outcomes in patients with acute myocardial infarction (AMI) [15, 28, 44]. A recent study compared NHR with MHR, suggesting the potential of NHR as a marker of coronary artery stenosis [45]. In another study, Kou et al. identified NHR as a significant factor associated with coronary artery stenosis and an independent predictor of severe cases [12]. Pan et al. reported a significant correlation between NHR and both cardiac ultrasound parameters and cardiovascular risk in a healthy population. They suggested that NHR could be a valuable marker for the early detection and management of cardiovascular disease in this group [46]. Jiang et al. reported that a higher NHR was independently associated with all‐cause and cardiovascular mortality in the general population [30]. The findings of these studies were consistent with our findings.

It should also be noted that the Gensini score does not necessarily reflect plaque inflammation or vulnerability. Many acute coronary events arise from biologically active, nonobstructive plaques, and advanced CT‐based techniques, such as plaque characterization and assessment of peri coronary adipose tissue attenuation, can provide information on plaque composition and vascular inflammation that is not captured by angiography [47]. Therefore, our findings should not be interpreted as evidence that NHR reflects plaque inflammation or vulnerability.

## 5. Limitations

This study has several limitations. First, the small sample size, particularly in the non‐CAD group, limited the precision of estimates; therefore, Model III was designated as the primary model, whereas Model IV was used for sensitivity analysis. Second, NHR was measured only at admission, precluding assessment of temporal changes. Third, the single‐center, retrospective cross‐sectional design, selection of patients referred for invasive CA, and exclusion of 163 patients with incomplete records may limit generalizability and introduce selection bias. Fourth, other inflammatory markers were not assessed, and comparisons with the NLR were not possible. Fifth, CA cannot characterize plaque composition or inflammation. Finally, clinical outcomes were not evaluated, and the proposed NHR cut‐off values lacked external validation. Thus, these findings should be considered preliminary, and larger, multicenter prospective studies are needed to confirm the associations and assess their clinical relevance.

## 6. Conclusion

This study provides evidence that the NHR is significantly associated with both the presence and severity of CAD in patients undergoing CA. Elevated NHR levels were observed in patients with CAD and were progressively higher in those with greater Gensini scores, suggesting a relationship between NHR and the angiographic extent of coronary atherosclerosis. However, NHR showed only modest discriminative ability, and its incremental value, prognostic significance, and clinical utility remain to be established. Future studies with larger and more diverse populations are needed to validate these findings and explore the mechanisms underlying the relationship between NHR and CAD pathophysiology.

## Author Contributions

Hamidreza Mohammadi: conceptualization; methodology; software; formal analysis; visualization; project administration; writing—original draft; writing—review and editing. Abbas Andishmand: conceptualization; methodology; supervision; writing—review and editing. Faezeh Badkoubeh: validation; writing—original draft; writing—review and editing. Amin Salehi‐Abargouei: investigation; supervision; writing—review and editing. Mohammadtaghi Sarebanhassanabadi: investigation; supervision; project administration; writing—review and editing. Danial Chaleshi: writing—review and editing. Marzieh Azimizadeh: data curation; formal analysis; visualization; project administration; writing—original draft; writing—review and editing. Matthew Budoff: supervision; writing—review and editing. Ladan Yaghmaei: data curation; writing—original draft; writing—review and editing. Farzaneh‐Sadat Mirjalili: data curation; writing—review and editing. Amin Entezari: data curation; writing—review and editing. Faezeh Golvardi‐Yazdi: writing—review and editing. Mohammadreza Rafiei: data curation; resources; writing—review and editing.

## Funding

No funding was received for this manuscript.

## Disclosure

The lead author Marzieh Azimizadeh affirms that this manuscript is an honest, accurate, and transparent account of the study being reported; that no important aspects of the study have been omitted; and that any discrepancies from the study as planned (and, if relevant, registered) have been explained. All authors have read and approved the final version of the manuscript. Marzieh Azimizadeh had full access to all of the data in this study and takes complete responsibility for the integrity of the data and the accuracy of the data analysis.

## Ethics Statement

The study was approved by the ethics committee of Shahid Sadoughi University of Medical Sciences (IR.SSU.REC.1402.006) and conducted based on the Declaration of Helsinki on medical research.

## Consent

Informed consent was obtained from eligible individuals.

## Conflicts of Interest

The authors declare no conflicts of interest.

## Supporting information


**Supporting Information** Additional supporting information can be found online in the Supporting Information section. Table S1: Fully adjusted multivariable logistic regression model (Model IV) for CAD and a high Gensini score, presenting odds ratios and 95% confidence intervals for NHR and all covariates. Table S2: Bootstrap stability analysis (1000 resamples) of the association between NHR and CAD and a high Gensini score in the primary (Model III) and fully adjusted (Model IV) models.

## Data Availability

The data that support the findings of this study are available from the corresponding author upon reasonable request.

## References

[1] Ralapanawa U. and Sivakanesan R. , Epidemiology and the Magnitude of Coronary Artery Disease and Acute Coronary Syndrome: A Narrative Review, Journal of Epidemiology and Global Health. (2021) 11, no. 2, 169–177, 10.2991/jegh.k.201217.001, 33605111. PMC8242111 33605111

[2] Duggan J. , Peters A. , Trachiotis G. , and Antevil J. , Epidemiology of Coronary Artery Disease, Surgical Clinics of North America. (2022) 102, no. 3, 499–516, 10.1016/j.suc.2022.01.007. 35671770

[3] Sanchis-Gomar F. , Pérez-Quilis C. , Leischik R. , and Lucia A. , Epidemiology of Coronary Heart Disease and Acute Coronary Syndrome, Annals of Translational Medicine. (2016) 4, no. 13, 10.21037/atm.2016.06.33, 27500157. PMC4958723 27500157

[4] Sarebanhassanabadi M. , Mirjalili S. , Marques-Vidal P. , Kraemer A. , and Namayandeh S. , Coronary Artery Disease Incidence, Risk Factors, Awareness, and Medication Utilization in a 10-Year Cohort Study, BMC Cardiovascular Disorders. (2024) 24, no. 1, 10.1186/s12872-024-03769-3, 38347457. PMC10863377 38347457

[5] Heidari-Foroozan M. , Farshbafnadi M. , Golestani A. , Younesian S. , Jafary H. , Rashidi M. , Tabatabaei-Malazy O. , Rezaei N. , Kheirabady M. M. , Ghotbi A. B. , and Ghamari S. H. , National and Subnational Burden of Cardiovascular Diseases in Iran from 1990 to 2021: Results From Global Burden of Diseases 2021 Study, Global Heart. (2025) 20, no. 1, 10.5334/gh.1429. PMC12083073 40386247

[6] Li X. , Li J. , and Wu G. , Relationship of Neutrophil-to-Lymphocyte Ratio With Carotid Plaque Vulnerability and Occurrence of Vulnerable Carotid Plaque in Patients With Acute Ischemic Stroke, BioMed Research International. (2021) 2021, 6894623, 10.1155/2021/6894623, 34250090. PMC8238559 34250090

[7] Chalela J. , Evaluating the Carotid Plaque: Going Beyond Stenosis, Cerebrovascular Diseases. (2009) 27, no. Supplement 1, 19–24, 10.1159/000200438, 19342830. 19342830

[8] Güven R. , Akyol K. C. , Bayar N. , Güngör F. , AKcA A. H. , and Çelik A. , Neutrophil Count as a Predictor of Critical Coronary Artery Stenosis in Young Patients, Iranian Journal of Public Health. (2018) 47, no. 5, 765–767, 29922624. PMC6005979 29922624

[9] Parhofer K. G. , Increasing HDL-Cholesterol and Prevention of Atherosclerosis: A Critical Perspective, Atherosclerosis Supplements. (2015) 18, 109–111, 10.1016/j.atherosclerosissup.2015.02.020, 25936313. 25936313

[10] Aggarwal J. , Kathariya M. G. , and Verma P. K. , LDL-C, NON-HDL-C and APO-B for Cardiovascular Risk Assessment: Looking for the Ideal Marker, Indian Heart Journal. (2021) 73, no. 5, 544–548, 10.1016/j.ihj.2021.07.013, 34627566. PMC8514398 34627566

[11] Oram J. F. and Heinecke J. W. , ATP-Binding Cassette Transporter A1: A Cell Cholesterol Exporter That Protects Against Cardiovascular Disease, Physiological Reviews. (2005) 85, no. 4, 1343–1372, 10.1152/physrev.00005.2005, 16183915. 16183915

[12] Kou T. , Luo H. , and Yin L. , Relationship Between Neutrophils to HDL-C Ratio and Severity of Coronary Stenosis, BMC Cardiovascular Disorders. (2021) 21, no. 1, 1–8, 10.1186/s12872-020-01771-z. PMC7936429 33676400

[13] Oleksiak A. , Kępka C. , Rucińska K. , Marcinkiewicz K. , Demkow M. , and Kruk M. , High-Density Lipoprotein Cholesterol, Triglycerides, and Characteristics of Coronary Atherosclerosis in Patients With Significant Coronary Artery Disease Newly Diagnosed by Computed Tomography Coronary Angiography, Kardiologia Polska (Polish Heart Journal). (2023) 81, no. 3, 273–280, 10.33963/KP.a2022.0279, 36475513. 36475513

[14] Yılmaz S. , Canpolat U. , Başer K. , Ünal S. , Kuyumcu M. S. , and Aydoğdu S. , Neutrophil-to-Lymphocyte Ratio Predicts Functionally Significant Coronary Artery Stenosis in Patients With Stable Coronary Artery Disease, Archives of the Turkish Society of Cardiology/Türk Kardiyoloji Derneği Arşivi. (2017) 46, no. 2, 10.5543/tkda.2017.16709. 29512613

[15] Lamichhane P. , Agrawal A. , Abouainain Y. , Abousahle S. , and Regmi P. R. , Utility of Neutrophil-to-high-Density Lipoprotein-Cholesterol Ratio in Patients With Coronary Artery Disease: A Narrative Review, Journal of International Medical Research. (2023) 51, no. 4, 03000605231166518, 10.1177/03000605231166518, 37038922. PMC10107976 37038922

[16] Golabchi A. , Gharakhani D. , Baghbeheshti M. , Gilasi H. , Jafari A. , Sadeghi A. M.-M. , Mohammadi H. , and Mirhosseini S. M. , Inflammatory Biomarkers as Predictors of Calcific Aortic Stenosis Severity: Examining Platelet to Lymphocyte and Neutrophil to Lymphocyte Ratios, Cardiovascular Biomedicine Journal. (2023) 3, no. 2, 18–24, 10.18502/cbj.v3i2.15174.

[17] Gao J. , Lu J. , Sha W. , Xu B. , Zhang C. , Wang H. , Xia J. , Zhang H. , Tang W. , and Lei T. , Relationship Between the Neutrophil to High-Density Lipoprotein Cholesterol Ratio and Severity of Coronary Artery Disease in Patients With Stable Coronary Artery Disease, Frontiers in Cardiovascular Medicine. (2022) 9, 10.3389/fcvm.2022.1015398. PMC9729241 36505389

[18] Motallaei M. , Darand M. , Taftian M. , Beigrezaei S. , Golvardi-Yazdi F. , Mohyadini M. , Mirjalili F. , Darabi Z. , Ahmadi Vasmehjani A. , Seyedhosseini S. , Sareban-Hassanabadi M. T. , Khayyatzadeh S. S. , and Salehi-Abargouei A. , A Cross- Sectional Study on the Association Between Lifestyle Factors and Coronary Artery Stenosis Severity Among Adults Living in Central Iran: A Protocol for the Iranian- CARDIO Study, ARYA Atherosclerosis. (2024) 20, no. 1, 51–61, 10.48305/arya.2023.41026.2843, 39165856. PMC11331555 39165856

[19] Cockcroft D. W. and Gault H. , Prediction of Creatinine Clearance From Serum Creatinine, Nephron. (1976) 16, no. 1, 31–41, 1244564. 10.1159/000180580 1244564

[20] Avci A. , Fidan S. , Tabakçı M. M. , Toprak C. , Alizade E. , Acar E. , Bayam E. , Tellice M. , Naser A. , and Kargın R. , Association Between the Gensini Score and Carotid Artery Stenosis, Korean Circulation Journal. (2016) 46, no. 5, 639–645, 10.4070/kcj.2016.46.5.639, 27721854. PMC5054175 27721854

[21] Mirjalili F.-S. , Baghiani T. , Badkoubeh F. , Andishmand A. , Sarebanhassanabadi M. , Mohammadi H. , Salehi-Abargouei A. , Motallaei M. , and Seyedhosseini S. M. , Noninvasive Prediction of Coronary Artery Disease Severity: Comparative Analysis of Electrocardiographic Findings and Risk Factors With SYNTAX and Gensini Score, Science Progress. (2025) 108, no. 1, 00368504241309454, 10.1177/00368504241309454, 39781616. PMC11713963 39781616

[22] Karadeniz F. Ö. and Altuntaş E. , Correlation Between Frontal QRS-T Angle, Tp-e Interval, and Tp-e/QT Ratio to Coronary Artery Severity Assessed With SYNTAX Score in Stable Coronary Artery Disease Patients, Journal of Arrhythmia. (2022) 38, no. 5, 783–789, 10.1002/joa3.12756, 36237856. PMC9535749 36237856

[23] Khan M. A. , Hashim M. J. , Mustafa H. , Baniyas M. Y. , Al Suwaidi S. K. B. M. , AlKatheeri R. , Alblooshi F. M. , Almatrooshi M. E. , Alzaabi M. E. , Al Darmaki R. S. , and Lootah S. N. , Global Epidemiology of Ischemic Heart Disease: Results From the Global Burden of Disease Study, Cureus. (2020) 12, no. 7, 10.7759/cureus.9349, 32742886. PMC7384703 32742886

[24] Body R. , Carley S. , McDowell G. , Jaffe A. S. , France M. , Cruickshank K. , Wibberley C. , Nuttall M. , and Mackway-Jones K. , Rapid Exclusion of Acute Myocardial Infarction in Patients With Undetectable Troponin Using a High-Sensitivity Assay, Journal of the American College of Cardiology. (2011) 58, no. 13, 1332–1339, 10.1016/j.jacc.2011.06.026, 21920261. 21920261

[25] Mor-Avi V. , Patel M. B. , Maffessanti F. , Singh A. , Medvedofsky D. , Zaidi S. J. , Mediratta A. , Narang A. , Nazir N. , Kachenoura N. , Lang R. M. , and Patel A. R. , Fusion of Three-Dimensional Echocardiographic Regional Myocardial Strain With Cardiac Computed Tomography for Noninvasive Evaluation of the Hemodynamic Impact of Coronary Stenosis in Patients With Chest Pain, Journal of the American Society of Echocardiography. (2018) 31, no. 6, 664–673, 10.1016/j.echo.2018.01.019, 29576220. PMC5988992 29576220

[26] Wang Q. , Jing H. , Lin J. , Wu Z. , Tian Y. , Gong K. , Guo Q. , Yang X. , Wang L. , Li Z. , and Duan Y. , Programmed Prodrug Breaking the Feedback Regulation of P-Selectin in Plaque Inflammation for Atherosclerotic Therapy, Biomaterials. (2022) 288, 121705, 10.1016/j.biomaterials.2022.121705, 36002347. 36002347

[27] Incalcaterra E. , Accardi G. , Balistreri C. R. , Caimi G. , Candore G. , Caruso M. , and Caruso C. , Pro-Inflammatory Genetic Markers of Atherosclerosis, Current Atherosclerosis Reports. (2013) 15, no. 6, 1–9, 10.1007/s11883-013-0329-5. 23591672

[28] Huang J.-B. , Chen Y.-S. , Ji H.-Y. , Xie W.-M. , Jiang J. , Ran L.-S. , Zhang C. T. , and Quan X. Q. , Neutrophil to High-Density Lipoprotein Ratio Has a Superior Prognostic Value in Elderly Patients With Acute Myocardial Infarction: A Comparison Study, Lipids in Health and Disease. (2020) 19, 1–12. 10.1186/s12944-020-01238-2 PMC7126405 32247314

[29] Makrygiannis S. S. , Ampartzidou O. S. , Zairis M. N. , Patsourakos N. G. , Pitsavos C. , Tousoulis D. , Prekates A. A. , Foussas S. G. , and Cokkinos D. V. , Prognostic Usefulness of Serial C-Reactive Protein Measurements in ST-Elevation Acute Myocardial Infarction, American Journal of Cardiology. (2013) 111, no. 1, 26–30, 10.1016/j.amjcard.2012.08.041, 23040593. 23040593

[30] Jiang M. , Sun J. , Zou H. , Li M. , Su Z. , Sun W. , and Kong X. , Prognostic Role of Neutrophil to High-Density Lipoprotein Cholesterol Ratio for All-Cause and Cardiovascular Mortality in the General Population, Frontiers in Cardiovascular Medicine. (2022) 9, 807339, 10.3389/fcvm.2022.807339, 35211525. PMC8861276 35211525

[31] Wang Y. , Zhang J. , Li H. , Kong W. , Zheng J. , Li Y. , Wei Q. , Li Q. , Yang L. , Xu Y. , Li L. , Wang H. , Sun H. , Xia W. , Liu G. , Zhong X. , Qiu K. , Wang H. , Liu H. , Song X. , Xiong S. , Liu Y. , Cui Z. , Chen L. , and Zeng T. , Prognostic Value of Leucocyte to High-Density Lipoprotein-Cholesterol Ratios in COVID-19 Patients and the Diabetes Subgroup, Frontiers in Endocrinology. (2021) 12, 727419, 10.3389/fendo.2021.727419, 34589058. PMC8473871 34589058

[32] Ren H. , Zhu B. , Zhao Z. , Li Y. , Deng G. , Wang Z. , Ma B. , Feng Y. , Zhang Z. , Zhao X. , Ali Sheikh M. S. , and Xia K. , Neutrophil to High-Density Lipoprotein Cholesterol Ratio as the Risk Mark in Patients With Type 2 Diabetes Combined With Acute Coronary Syndrome: A Cross-Sectional Study, Scientific Reports. (2023) 13, no. 1, 10.1038/s41598-023-35050-6, 37188740. PMC10185574 37188740

[33] Murphy A. J. , Woollard K. J. , Suhartoyo A. , Stirzaker R. A. , Shaw J. , Sviridov D. , and Chin-Dusting J. P. , Neutrophil Activation is Attenuated by High-Density Lipoprotein and Apolipoprotein A-I in In Vitro and In Vivo Models of Inflammation, Arteriosclerosis, Thrombosis, and Vascular Biology. (2011) 31, no. 6, 1333–1341, 10.1161/ATVBAHA.111.226258, 21474825. 21474825

[34] Chistiakov D. A. , Grechko A. V. , Myasoedova V. A. , Melnichenko A. A. , and Orekhov A. N. , The Role of Monocytosis and Neutrophilia in Atherosclerosis, Journal of Cellular and Molecular Medicine. (2018) 22, no. 3, 1366–1382, 10.1111/jcmm.13462, 29364567. PMC5824421 29364567

[35] Soehnlein O. , Multiple Roles for Neutrophils in Atherosclerosis, Circulation Research. (2012) 110, no. 6, 875–888, 10.1161/CIRCRESAHA.111.257535. 22427325

[36] Pertiwi K. R. , van der Wal A. C. , Pabittei D. R. , Mackaaij C. , van Leeuwen M. B. , Li X. , and de Boer O. J. , Neutrophil Extracellular Traps Participate in All Different Types of Thrombotic and Haemorrhagic Complications of Coronary Atherosclerosis, Thrombosis and Haemostasis. (2018) 118, no. 6, 1078–1087, 10.1055/s-0038-1641749. 29672788

[37] Dragu R. , Huri S. , Zukermann R. , Suleiman M. , Mutlak D. , Agmon Y. , Kapeliovich M. , Beyar R. , Markiewicz W. , Hammerman H. , and Aronson D. , Predictive Value of White Blood Cell Subtypes for Long-Term Outcome Following Myocardial Infarction, Atherosclerosis. (2008) 196, no. 1, 405–412, 10.1016/j.atherosclerosis.2006.11.022, 17173924. 17173924

[38] Chen J. , Chen M.-H. , Li S. , Guo Y.-L. , Zhu C.-G. , Xu R.-X. , Zhang Y. , Sun J. , Qing P. , Liu G. , and Li J. J. , Usefulness of the Neutrophil-to-Lymphocyte Ratio in Predicting the Severity of Coronary Artery Disease: A Gensini Score Assessment, Journal of atherosclerosis and Thrombosis. (2014) 21, no. 12, 1271–1282, 10.5551/jat.25940, 25069816. 25069816

[39] Gomaraschi M. , Calabresi L. , and Franceschini G. , Protective Effects of HDL Against Ischemia/Reperfusion Injury, Frontiers in Pharmacology. (2016) 7, 10.3389/fphar.2016.00002. PMC4725188 26834639

[40] Pérez-Morga D. , Vanhollebeke B. , Paturiaux-Hanocq F. , Nolan D. P. , Lins L. , Homblé F. , Vanhamme L. , Tebabi P. , Pays A. , Poelvoorde P. , and Jacquet A. , Apolipoprotein L-I Promotes Trypanosome Lysis by Forming Pores in Lysosomal Membranes, Science. (2005) 309, no. 5733, 469–472, 10.1126/science.1114566, 16020735. 16020735

[41] Okajima F. , Plasma lipoproteins Behave as Carriers of Extracellular Sphingosine 1-Phosphate: Is This an Atherogenic Mediator or an Anti-Atherogenic Mediator?, Lipids. (2002) 1582, no. 1-3, 132–137, 10.1016/S1388-1981(02)00147-6. 12069820

[42] Jia C. , Anderson J. L. , Gruppen E. G. , Lei Y. , Bakker S. J. , Dullaart R. P. , and Tietge U. J. , High-Density Lipoprotein Anti-Inflammatory Capacity and Incident Cardiovascular Events, Circulation. (2021) 143, no. 20, 1935–1945, 10.1161/CIRCULATIONAHA.120.050808, 33840204. 33840204

[43] Smith C. K. , Vivekanandan-Giri A. , Tang C. , Knight J. S. , Mathew A. , Padilla R. L. , Gillespie B. W. , Carmona-Rivera C. , Liu X. , Subramanian V. , Hasni S. , Thompson P. R. , Heinecke J. W. , Saran R. , Pennathur S. , and Kaplan M. J. , Neutrophil Extracellular Trap–Derived Enzymes Oxidize High‐Density Lipoprotein: An Additional Proatherogenic Mechanism in Systemic Lupus Erythematosus, Arthritis & Rheumatology. (2014) 66, no. 9, 2532–2544, 10.1002/art.38703, 24838349. PMC4146708 24838349

[44] Ozgeyik M. and Ozgeyik M. O. , Long-Term Prognosis After Treatment of Total Occluded Coronary Artery Is Well Predicted by Neutrophil to High-Density Lipoprotein Ratio: A Comparison Study, Kardiologiia. (2021) 61, no. 7, 60–67, 10.18087/cardio.2021.7.n1637, 34397343. 34397343

[45] Guo J. , Chen M. , Hong Y. , Huang Y. , Zhang H. , Zhou Y. , Zhou B. , and Fu M. , Comparison of the Predicting Value of Neutrophil to High-Density Lipoprotein Cholesterol Ratio and Monocyte to High-Density Lipoprotein Cholesterol Ratio for In-Hospital Prognosis and Severe Coronary Artery Stenosis in Patients With ST-Segment Elevation Acute Myocardial Infarction Following Percutaneous Coronary Intervention: A Retrospective Study, Journal of Inflammation Research. (2023) 16, 4541–4557, 10.2147/JIR.S425663. PMC10588721 37868828

[46] Pan X. , Zhang X. , Ban J. , Yue L. , Ren L. , and Chen S. , Association of Neutrophil to High-Density Lipoprotein Cholesterol Ratio With Cardiac Ultrasound Parameters and Cardiovascular Risk: A Cross-Sectional Study Based on Healthy Populations, Journal of Inflammation Research. (2023) 16, 1853–1865, 10.2147/JIR.S406102, 37138930. PMC10150755 37138930

[47] Mátyás B. B. , Benedek I. , Rat N. , Gerculy R. , and Benedek T. , Coronary Plaque Vulnerability and Pericoronary Adipose Tissue Inflammation: Emerging Insights From Advanced CT Imaging, Medicina. (2026) 62, no. 4, 10.3390/medicina62040630, 42075502. PMC13117169 42075502

