# Supplementary material for: Association of the Neutrophil‐to‐HDL‐C Ratio With Coronary Artery Disease and Coronary Stenosis Severity in Patients Undergoing Coronary Angiography: Results From the Iranian‐CARDIO Study
Source: Cardiovasc Ther. 2026 Nov 10;2026:3017486. doi: 10.1155/cdr/3017486 (PMC13752560; doi:10.1155/cdr/3017486)
Supplement: Supplementary file 1 [file CDR-2026-3017486-s001.docx]

**Supplementary Material**

**Supplementary Table S1.** Fully adjusted multivariable logistic regression model (Model IV) for CAD and a high Gensini score.

|  | **CAD** | | **High Gensini** | |
| --- | --- | --- | --- | --- |
| **Variable** | **OR (95% CI)** | **P-value** | **OR (95% CI)** | **P-value** |
| NHR (per 1 SD) | 2.31 (1.38–3.84) | 0.001 | 1.62 (1.13–2.31) | 0.008 |
| Age (per year) | 1.08 (1.03–1.13) | 0.001 | 1.05 (1.01–1.09) | 0.024 |
| Male sex | 4.34 (1.82–10.34) | <0.001 | 2.85 (1.23–6.64) | 0.015 |
| Smoking | 1.83 (0.66–5.06) | 0.245 | 1.12 (0.54–2.30) | 0.766 |
| BMI (per kg/m²) | 0.97 (0.89–1.06) | 0.500 | 1.03 (0.95–1.11) | 0.531 |
| SBP (per mmHg) | 1.015 (0.988–1.043) | 0.278 | 1.019 (0.995–1.045) | 0.124 |
| DBP (per mmHg) | 0.974 (0.930–1.020) | 0.256 | 0.973 (0.938–1.010) | 0.149 |
| FBS (per mg/dL) | 1.008 (0.996–1.020) | 0.176 | 1.003 (0.996–1.009) | 0.431 |
| TC (per mg/dL) | 1.001 (0.997–1.005) | 0.711 | 1.000 (0.997–1.004) | 0.778 |
| TG (per mg/dL) | 1.000 (0.995–1.006) | 0.903 | 1.002 (0.998–1.006) | 0.362 |
| LDL-C (per mg/dL) | 1.004 (0.991–1.017) | 0.571 | 1.004 (0.994–1.014) | 0.430 |
| AST (per U/L) | 1.002 (0.984–1.020) | 0.862 | 1.009 (0.995–1.023) | 0.189 |
| ALT (per U/L) | 1.005 (0.997–1.013) | 0.205 | 0.998 (0.995–1.002) | 0.442 |
| eGFR (per mL/min) | 0.998 (0.986–1.009) | 0.677 | 1.003 (0.994–1.013) | 0.485 |
| Urea (per mg/dL) | 0.955 (0.921–0.990) | 0.013 | 1.010 (0.976–1.044) | 0.579 |
| DM | 1.35 (0.54–3.43) | 0.522 | 1.02 (0.48–2.20) | 0.956 |
| HTN | 1.24 (0.58–2.68) | 0.581 | 1.12 (0.57–2.20) | 0.748 |

ORs for NHR are per 1-SD increase (1 SD = 1.71). NHR was calculated as the neutrophil count (×10³/µL) divided by HDL-C in mmol/L (HDL-C in mg/dL × 0.02586). High Gensini: highest Gensini tertile (n = 64) vs. all other patients (n = 186). Abbreviations: ALT, alanine aminotransferase; AST, aspartate aminotransferase; BMI, body mass index; CAD, coronary artery disease; CI, confidence interval; DBP, diastolic blood pressure; DM, diabetes mellitus; eGFR, estimated glomerular filtration rate; FBS, fasting blood sugar; HTN, hypertension; LDL-C, low-density lipoprotein cholesterol; NHR, neutrophil-to-HDL-C ratio; OR, odds ratio; SBP, systolic blood pressure; SD, standard deviation; TC, total cholesterol; TG, triglycerides.

**Supplementary Table S2.** Bootstrap stability of the association between NHR (per 1-SD increase) and CAD and a high Gensini score in the primary (Model III) and fully adjusted (Model IV) models.

| **Outcome / model** | **Original OR (95% CI)** | **Bootstrap median OR** | **Bootstrap percentile 95% CI** | **Replicates with OR > 1 (%)** |
| --- | --- | --- | --- | --- |
| **CAD** |  |  |  |  |
| Model III (primary) | 2.01 (1.28–3.14) | 2.04 | 1.28–3.58 | 99.8 |
| Model IV (sensitivity) | 2.31 (1.38–3.84) | 2.43 | 1.28–5.39 | 99.9 |
| **High Gensini score** |  |  |  |  |
| Model III (primary) | 1.56 (1.14–2.12) | 1.59 | 1.15–2.29 | 100.0 |
| Model IV (sensitivity) | 1.62 (1.13–2.31) | 1.71 | 1.11–2.91 | 99.4 |

Nonparametric bootstrap with 1,000 resamples of the full sample (n = 250) drawn with replacement; all models converged in every replicate. Original estimates are maximum-likelihood ORs with Wald 95% CIs; bootstrap CIs are the 2.5th and 97.5th percentiles of the bootstrap distribution of the NHR coefficient. ORs are per 1-SD increase in NHR (1 SD = 1.71). Model III: adjusted for age, sex, smoking, BMI, SBP, and DBP. Model IV: additionally adjusted for FBS, TC, TG, LDL-C, AST, ALT, eGFR, urea, DM, and HTN. High Gensini: highest Gensini tertile (n = 64) vs. all other patients (n = 186). Abbreviations as in Supplementary Table S1.
